# MENA Is a Transcriptional Target of the Wnt/Beta-Catenin Pathway

**DOI:** 10.1371/journal.pone.0037013

**Published:** 2012-05-17

**Authors:** Ayaz Najafov, Tuncay Şeker, İpek Even, Gerta Hoxhaj, Osman Selvi, Duygu Esen Özel, Ahmet Koman, Necla Birgül-İyison

**Affiliations:** 1 Department of Molecular Biology and Genetics, Bogazici University, Istanbul, Turkey; 2 MRC Phosphorylation Unit, University of Dundee, Dundee, Scotland, United Kingdom; 3 Department of Molecular and Developmental Genetics, Vlaams Instituut voor Biotechnologie, Leuven, Belgium; University of Birmingham, United Kingdom

## Abstract

Wnt/β-catenin signalling pathway plays important roles in embryonic development and carcinogenesis. Overactivation of the pathway is one of the most common driving forces in major cancers such as colorectal and breast cancers. The downstream effectors of the pathway and its regulation of carcinogenesis and metastasis are still not very well understood. In this study, which was based on two genome-wide transcriptomics screens, we identify *MENA* (*ENAH*, Mammalian enabled homologue) as a novel transcriptional target of the Wnt/β-catenin signalling pathway. We show that the expression of *MENA* is upregulated upon overexpression of degradation-resistant β-catenin. Promoters of all mammalian *MENA* homologues contain putative binding sites for Tcf4 transcription factor – the primary effector of the Wnt/β-catenin pathway and we demonstrate functionality of these Tcf4-binding sites using luciferase reporter assays and overexpression of β-catenin, Tcf4 and dominant-negative Tcf4. In addition, lithium chloride-mediated inhibition of GSK3β also resulted in increase in *MENA* mRNA levels. Chromatin immunoprecipitation showed direct interaction between β-catenin and *MENA* promoter in Huh7 and HEK293 cells and also in mouse brain and liver tissues. Moreover, overexpression of Wnt1 and Wnt3a ligands increased *MENA* mRNA levels. Additionally, knock-down of *MENA* ortholog in D. melanogaster *eyeful* and *sensitized* eye cancer fly models resulted in increased tumor and metastasis formations. In summary, our study identifies MENA as novel nexus for the Wnt/β-catenin and the Notch signalling cascades.

## Introduction

Wnt/β-catenin signalling pathway is critical for early and late embryonic development [1], [2] and it plays important roles during tumorigenesis of various cancers [3]. β-catenin is a multifunctional adaptor protein/transcription factor that is deregulated in many cancers. In the absence of Wnt ligand, cytosolic β-catenin levels are down-regulated via a degradation complex including CK1α, GSK3β, Axin, APC, and PP2A in which processive phosphorylation of β-catenin by CK1α and GSK3β leads to its ubiquitination and proteasomal degradation. In the presence of Wnt ligand, upon its binding to the frizzled/LRP5/6 receptor complex, Dishevelled (Dsh/Dvl) is activated, at least in part by phosphorylation. Activated Dsh is part of a protein complex that recruits GSK3β away from the β-catenin degradation complex, allowing the dephosphorylation and nuclear import of β-catenin, where it activates the TCF/LEF family of transcriptional factors that control expression of various genes related to cell cycle and differentiation [4], [5].

Wnt/β-catenin pathway is strongly implicated in breast carcinogenesis, in addition to many other cancer types. Transgenic mice expressing degradation-resistant β-catenin in mammary gland tissue develop breast tumors [6], [7]. According to immuno-histochemical analysis, nuclear and cytoplasmic β-catenin levels have been found to be elevated in about 60% of the breast tumors [8], [9]. Furthermore, reduced levels of extracellular Wnt-inhibitory molecules sFRP1 and WIF1 have been linked to 80% and 60% of breast carcinomas [10], [11]. Additionally, β-catenin has been associated with epidermal growth factor receptor (EGFR) family members [12]–[16] and the stability of β-catenin and its TCF/LEF-activating function has been suggested to be regulated via tyrosine phosphorylation by the EGFR family [15], [17]–[19], which may be significant for breast carcinogenesis, since human epidermal growth factor receptor 2 (HER2) is overexpressed in about 30% of human breast tumors [20], [21]. Hence, identification of novel targets of Wnt/β-catenin pathway, serves an important purpose for cancer research field, particularly breast cancer, since target genes of the pathway are potential anti-cancer drug targets.

Many actin-associated proteins play important roles in carcinogenesis of various types of cancers [22]. *MENA* is an actin-regulatory protein that belongs to ENA/VASP protein family [23]. Members of this protein family are localized at the tips of protruding lamellipodia and filopodia and adhesion foci; and they are involved in control of cell motility and cell-cell adhesion, which are important subjects for development of metastatic potential [23]. Di Modugno *et al.* [24] showed that human *MENA* (*hMENA*) is overexpressed in ∼75% of primary breast cancers.

In our previous study [25], we employed SAGE (Serial Analysis of Gene Expression) and genome-wide microarray approaches to screen for novel Wnt/β-catenin pathway targets by overexpressing degradation-resistant S33Y-β-catenin [26] in Huh7 (hepatocellular carcinoma) cell lines, which lacks detectable nuclear endogenous β-catenin levels [27]. In these screens we found *MENA* to be differentially expressed and in this study we show that *MENA* is a transcriptional target of the Wnt/β-catenin pathway.

Wnt and Notch pathways have essential roles in development with well-studied crosstalks, yet their interplay in cancer is not well understood. In order to investigate the role of *MENA* in tumorigenesis, we tested whether knock-down of the *Drosophila* homolog of *MENA* (*Ena*) can affect tumor formation in the *D. melanogaster* eye cancer fly models “eyeful” and “sensitized”. Eyeful flies have a metastatic eye tumor phenotype induced by activated Notch signalling due to overexpression of the Notch ligand *Delta* and overexpression of polycomb genes *lola* and *pipsqueak*. Sensitized flies overexpress only Delta and have an increased eye size phenotype, but no metastatic tumors. Interestingly, we found that *Ena* knock-down increases tumor formation and metastasis in both fly models, indicating that *Ena* has a tumor suppressor role at a crosstalk between the Wnt/β-catenin and the Notch signalling cascades.

## Results

### Overexpression of β-catenin or Wnt ligands leads to increase in mRNA levels of MENA and other actin-associated proteins

**Figure 1:**
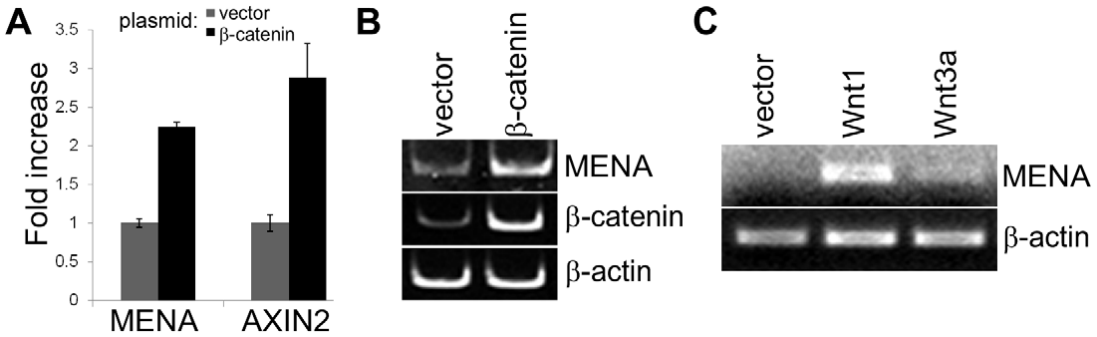
Overexpression of β-catenin-S33Y and Wnt ligans leads to increase in *MENA* transcription. (A) β-catenin-S33Y mutant was stably overexpressed in Huh7 cells and *MENA* and Axin2 mRNA levels were analyzed using qRT-PCR. (B) β-catenin-S33Y was transiently overexpressed (48 hrs) in U373 cells and *MENA* and β-catenin mRNA levels were compared using RT-PCR. (C) Wnt1 or Wnt3a ligans were overexpressed in Huh7 cells (48 hrs) and *MENA* mRNA levels were compared using RT-PCR.

This study stems from our previous work with Huh7 cells as a hepatocellular carcinoma model, in which SAGE and genome-wide microarray analysis were used to identify novel cancer markers related to Wnt/β-catenin pathway. This pathway is known to be relatively silent in Huh7 cells [27]. We generated stable Huh7 cell line overexpressing degradation-resistant β-catenin (S33Y mutation), as an approach to mimic activation of the Wnt/β-catenin pathway. This stable cell line was used for SAGE and microarray analyses. From these screens we found that *MENA* expression was upregulated 5.5-fold upon overexpression of the S33Y-β-catenin [25]. RT-PCR confirmed the microarray data (Fig. 1). Stable and transient overexpression of S33Y-β-catenin, as well as stimulation of the Wnt/β-catenin pathway by overexpression of the Wnt1 or Wnt3a ligands, in an attempt to stimulate the Wnt/β-catenin pathway through autocrine effect, increased *MENA* mRNA levels.

### TCF4-binding elements (TBEs) are well-conserved among MENA ortholog promoter sequences

**Figure 2:**
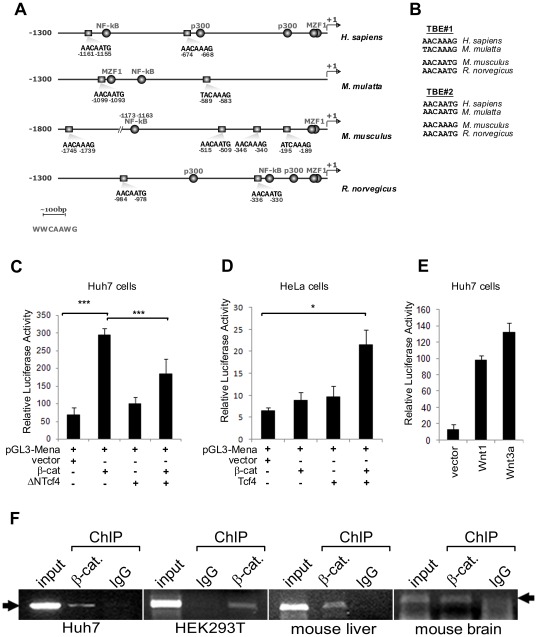
Promoters of mammalian *MENA* orthologs have functional Tcf4-binding elements (TBEs) that are regulated by the Wnt/β-catenin pathway. (A) Schematic representation of the promoters and the identified putative TBEs. The location of the TBEs is indicated by boxes and the sequences and locations with respect to transcriptional start sites (denoted by arrows) are shown under the boxes. Predicted binding motifs for other transcription factors are also indicated. The length of the bar is 100 bp. The WWCAAWG sequence is the consensus sequence for TBEs. (B) Alignment of proximal (TBE#1) and distal (TBE#2) TBE sequences. (C, D and E) Huh7 cells or HeLa cells were transfected with pGL3-*MENA* reporter plasmid and the promoter activity was stimulated by co-transfecting degradation-resistant β-catenin, Tcf4 or Wnt ligans or inhibited by co-transfecting dominant-negative Tcf4 (ΔNTcf4). The bars are given as averages (n = 3) normalized to Renilla luciferase activity, which was used an internal control. Error bars represent standard deviation (* = p<0.05, ** = p<0.01 and *** = p<0.005, Student's t-test). Each graph is a representative of at least two independent experiments. “β-cat” denotes the quadruple-mutant β-catenin-4m (See Materials & Methods). (F) Chromatin immunoprecipitation assay was done with monoclonal anti-β-catenin antibody and PCR (35–40 cycles) was used to detect *MENA* promoter fragments. β-catenin was found to interact with *MENA* promoter in Huh7 and HEK293T cell context, as well as in mouse brain tissue and liver tissue contexts. All experiments were repeated at least twice. An unrelated monoclonal antibody was used as the control IgG. The lower bands that appear in the mouse brain gel image are the primer-dimer bands. The arrow indicates the band of the expected size.

We analyzed the promoters of *Homo sapiens*, *Macaca mulatta*, *Mus musculus* and *Rattus norvegicus MENA* ortholog genes using Transcriptional Regulatory Element Database of Zhang Lab at CSHL (http://rulai.cshl.edu) and found two putative TBEs in all four of the promoters (Fig. 2A) with the exception of the mouse *MENA* promoter, which has four putative TBEs. Importantly, human and macaque TBEs proximal to the transcriptional start site (TSS) differed by only 1 nucleotide and the second TBEs, which were more distal to the TSS were identical (Fig. 2B). Also, the positions of the human and the macaque TBEs with respect to the TSS were found to be very similar. Equivocal sequence and position similarity between the mouse and the rat TBEs was also observed. All of the TBEs sequences matched the TCF4-binding consensus sequence 5′-WWCAAWG-3′.

### MENA promoter and β-catenin/TCF4 interact in vivo

To obtain further evidence for the interaction of *MENA* promoter with the β-catenin/TCF4 complex *in vivo*, we performed luciferase reporter assay utilizing the [−1250…+1] *MENA* 5′-upstream fragment as the reporter expression-driving promoter (Fig. 2C, 2D and 2E). We observed ∼5-fold stimulation of *MENA* promoter upon quadruple-mutant β-catenin overexpression in Huh7 cells, which was suppressed by co-overexpression of dominant-negative Tcf4 (ΔN-Tcf4) (Fig. 2C). In HeLa cells, the effect of β-catenin overexpression could only be observed when co-overexpressed with Tcf4 (Fig. 2D). Importantly, *MENA* promoter activity was stimulated by ∼6–9-fold upon overexpression of recombinant Wnt1, Wnt3a, Wnt4 and Wnt5a ligands in Huh7 cells (Fig. 2E). In order to test the physiologic relevance of the TBEs found in the *MENA* promoter, we performed chromatin immunoprecipitation assay with monoclonal anti-β-catenin antibody using Huh7 and HEK293 cells and mouse brain and liver tissues (Fig. 2F). PCR analysis of the DNA fragments immunoprecipitated with this antibody showed that *MENA* promoter and β-catenin/TCF4 complex interact *in vivo* in these cells and tissues.

### Inhibition of β-catenin degradation via lithium treatment leads to increased MENA mRNA level

**Figure 3:**
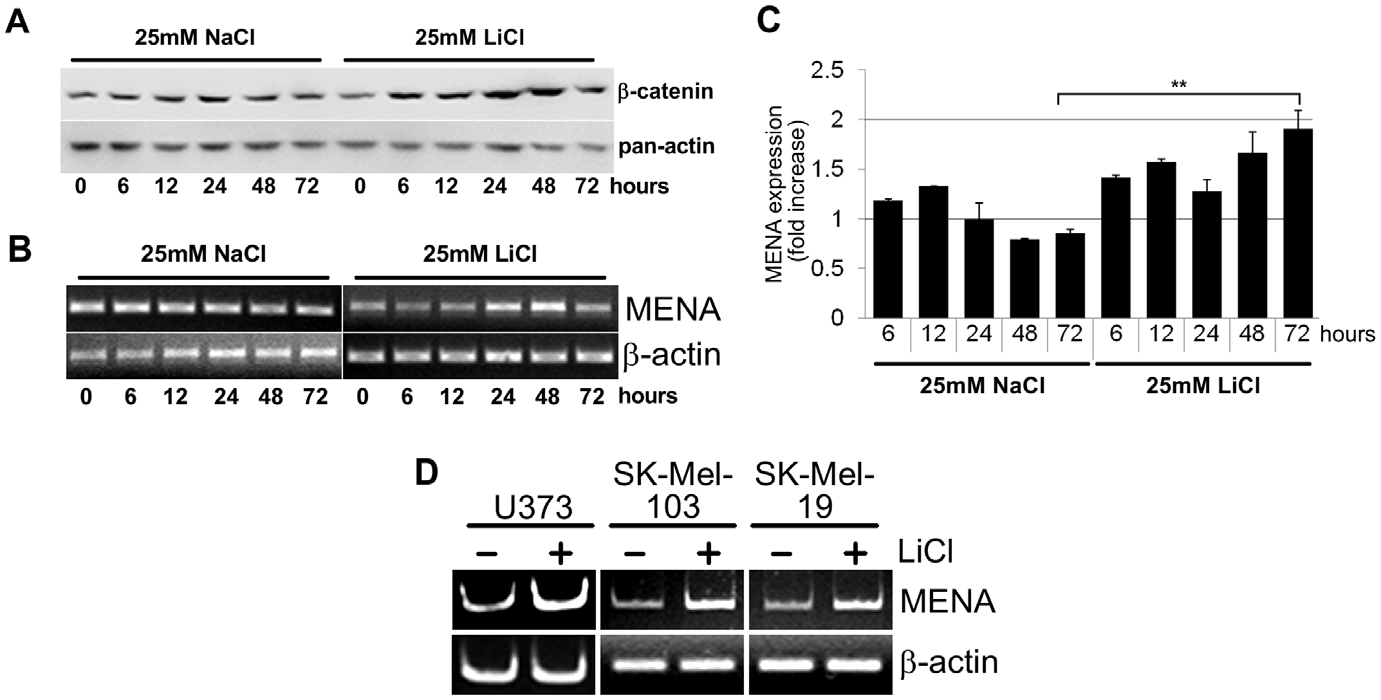
Lithium-mediated inhibition of GSK3β leads to β-catenin accumulation and increase in *MENA* mRNA levels. (A) Western blot analysis of Huh7 cells treated with either 25 mM NaCl or 25 mM LiCl for indicated timepoints. RT-PCR (B) and qRT-PCR (C) analysis of Huh7 cells treated with either 25 mM NaCl or 25 mM LiCl for indicated timepoints. (D) RT-PCR analysis of U373, Sk-Mel-103 and Sk-Mel-19 cells treated with LiCl for 24 hrs. Each image is a representative of at least two independent experiments.

Inhibition of GSK3β using LiCl is a widely-used approach to mimic an activated Wnt/β-catenin pathway. We treated cells with LiCl and observed the expected accumulation of endogenous β-catenin to be concomitant with a significant increase in *MENA* mRNA levels. In Huh7 cells, we performed a time-course of the LiCl treatment (Fig. 3A, 3B and 3C). Significant increase in *MENA* mRNA levels was observed after 24 hrs of LiCl-treatment. Huh7 cells entered senescence (data not shown) after 72 hrs of LiCl-treatment, which can be the reason for the drop in the β-catenin protein and *MENA* mRNA levels compared to the 48 hrs timepoint. Treatment of U373, Sk-Mel-103 and Sk-Mel-19 cells with LiCl for 24 hrs also lead to accumulation of endogenous β-catenin and increase in *MENA* mRNA levels (Fig. 3D).

### Knock-down of Ena in Drosophila induces tumor formation

**Figure 4:**
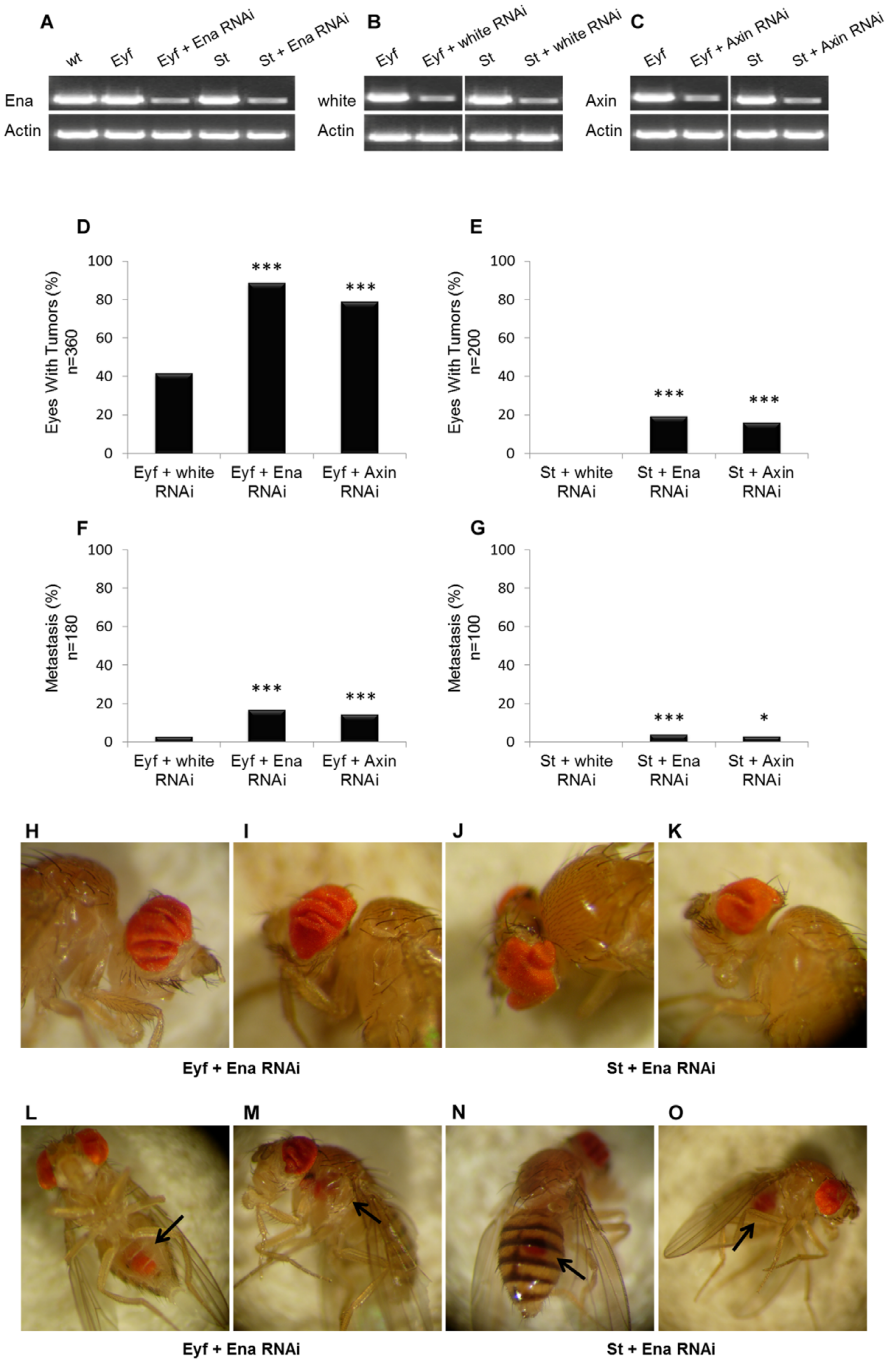
*Ena* knock-down induces tumor formation and metastasis *in vivo*. *Ena* (A), *white* (B) and *axin* (C) knock-down was confirmed by RT-PCR. Actin was used as an internal control. Significant increase of tumor formation (D and E) and metastasis (F and G) in eyeful (Eyf) (D, F) and sensitized (St) (E, G) flies upon knock-down of *ena* and *axin* genes is observed, when *white* knock-down is used as a control. (* indicates p<0.05; *** indicates p<0.001 as analyzed by the chi-square test). Representative images of tumor and metastasis formations in eyeful and sensitized flies upon knock-down of *Ena* (H-O). (H and I) ey-GAL4, UAS-Dl, eyeful/+; UAS-EnaRNAi/+ (“+”: wild-type chromosome) flies showing tumors in the eye tissue. Every fold in the eye tissue is counted as a single tumor. (J and K) ey-Gal4, UAS-Dl/+, UAS-EnaRNAi/+ flies with tumors in the eye. (L and M) ey-GAL4, UAS-Dl, eyeful/+; UAS-EnaRNAi/+ flies showing metastasis on the abdomen and thorax. (N and O) ey-Gal4, UAS-Dl/+, UAS-EnaRNAi/+ flies with metastasis on the back and abdomen.

To examine the role of *MENA* in tumorigenesis, we investigated whether downregulation of the Drosophila homolog of *MENA* gene *Ena* affects tumor formation in fly eye cancer models. Knock-down of *axin* in “eyeful” flies, which have high incidence of eye tumor formation, resulted in an increased frequency (79%) of eyes showing tumor formation and increased frequency (14%) of flies showing metastasis, compared to eyeful flies expressing a negative control RNAi (*white* gene) that showed only 42% of eyes with tumor formation and 8% of flies with metastases (Fig. 4D and 4F). Knock-down of *Ena* in eyeful flies resulted in 89% of tumor prevelance and resulted in 17% of flies with metastases (Fig. 4D, 4F, 4H–I and 4L–M).

When this experiment was conducted in “sensitized” flies, which have enlarged eyes, but no tumor formation, knock-down of *Ena* induced *de novo* tumor formation in 17% of eyes and metastasis formation in 4% of flies, compared to negative control (*white* gene) which did not result in any tumor or metastasis formation (Fig. 4E, 4G, 4J–K and 4N–O). Axin knock-down in sensitized flies resulted in comparable levels of tumor formation (16%) and metastasis (3%).

## Discussion

In this study, we have shown that *MENA*, a gene encoding an actin-regulatory protein, is a transcriptional target of the Wnt/β-catenin pathway. Overexpression of β-catenin and Wnt ligands resulted in increased *MENA* transcription (Fig. 1). Using luciferase reporter and chromatin immunoprecipitation assays, we confirmed that the putative TCF4-binding elements conserved among mammalian homologues of *MENA* are indeed functional and are regulated by the Wnt/β-catenin pathway (Fig. 2). Moreover, GSK3β inhibition by lithium treatment showed that *MENA* mRNA levels increase concurrently with the cytosolic accumulation of β-catenin (Fig. 3).

It is noteworthy that *MENA* is a Wnt/β-catenin pathway target, since *MENA* is overexpressed in ∼75% of breast cancers and the Wnt/β-catenin pathway is overactivated in many breast cancer cell lines and tumors. Moreover, recently, *MENA* was found to be overexpressed in colon cancer [28], [29] and Wnt/β-catenin pathway overactivation is one of the major causes of colon carcinomas. This suggests that MENA could be one of the downstream effectors of the Wnt/β-catenin pathway in these cancers. Importantly, Wnt/β-catenin pathway is known to regulate actin organization (e.g. via dishevelled) and formation of filopodia (via regulation of RhoU) [30], while *MENA* also an important component protruding filopodia.

Strikingly, *in vivo MENA* mRNA levels have been previously shown to be upregulated in the brains of lithium-treated mice [31]. The same study showed that *in vivo* β-catenin levels are also upregulated by this treatment. This is an important *in vivo* evidence for MENA being a Wnt/β-catenin pathway target in brain, since lithium-induced increased β-catenin levels lead to increase in transcription of the pathway targets.

In addition to its supposed role in carcinogenesis, *MENA* is known to be important in nervous system development, as *MENA*-null mice display subtle deficiencies in forebrain commissure formation [32]. Fascinatingly, Wnt pathways are known to regulate commissure formation (reviewed in ref. [33]) at least through Wnt4 and Wnt5.

In a recent genome-wide screen, *MENA* was found to be associated with schizophrenia [34]. It is intriguing that Wnt1, Fz3 and GSK3β – major Wnt/β-catenin pathway signalling components – have also been associated with schizophrenia or susceptibility to schizophrenia [35]–[37]. Thus, *MENA* could be one of the effectors of the Wnt signalling, abnormal activation of which is associated with the pathogenesis of the disease.

It is exciting that *MENA* promoter was found to be associated with β-catenin in a recent screen [38], where chromatin immunoprecipitation coupled with massively parallel sequencing (ChIP-Seq) was used to identify β-catenin binding regions in HCT116 human colon cancer cells, suggesting that *MENA* is a Wnt/β-catenin pathway target not only in liver and brain, but also in colon cancer contexts.

The Wnt and Notch signalling pathways play important roles in development and tumorigenesis and have reported crosstalks in these processes, which are not well understood. These two pathways have opposing effects during development. However, in tumorigenesis, there are many examples where Wnt and Notch pathways must be both activated to induce neoplasia, whereas there are only a few examples where these pathways contradict. For example, in epidermis, Notch1 is a tumour suppressor gene that represses the Wnt/β-catenin pathway [39], while Wnt signalling is known to promote epidermis neoplasia [40]. In this study, we find MENA to be a Wnt/β-catenin pathway target and show that it plays role in repressing Notch-mediated tumorigenesis in eye cancer fly models – a novel example of how Wnt and Notch pathways contradict in cancer. This is in contrast to the previously reported role of MENA as a tumorigenesis-promoting protein – MENA is overexpressed in ∼75% of primary breast cancers and has a role in activation of MAPK and Akt signalling [41], [42]. The discrepancy of our Drosophila results and the established upregulation of Mena in cancer can be due to the following: dysregulation of actin skeleton by Mena knock-down in *eyeful* and *sensitized* flies may lead to increased metastasis and neoplasia due to lack of proper cell attachment and increased cell motility, whereas high levels of Mena in many human cancers could be due to the fact that overexpression of Mena leads to overactivation of growth factor signalling and therefore cells overexpressing Mena would be predominant in certain tumors. In other words, artificially induced lack of cytoskeleton-related function of Mena (knock-down in flies) is promoting metastasis, whereas high levels of growth signalling-related function of Mena (in human cancers) result in increased proliferation and thus, increased neoplasia. Therefore, our findings put MENA at a critical point of crosstalk between Wnt/β-catenin and Notch pathways in regulating actin cytoskeleton and provide a novel example of a Wnt target that represses Notch signalling. Further studies are necessary to determine the role of MENA at the crossroads of Wnt and Notch signalling pathways, in the context of tumorigenesis and potentially brain development.

## Materials and Methods

### Cell Culture

All cell lines were obtained from American Tissue Culture Collection (http://www.atcc.org) and maintained in Dulbecco's modified Eagle's medium (Gibco) supplemented with 10% fetal bovine serum (Gibco) at 37°C in 5% CO_2_. Passages 6–15 were used for the experiments.

### Plasmids

5′-upstream fragments of h*MENA* gene were amplified from human genomic DNA using the following primers: “Forward”: [TAA GCT AGC GAA GTC ATC CCT ATA CCT AGT G] and “Reverse”: AAA AAG CTT CAT GGT GCC GGC GGC]. The PCR fragments were cloned into a Firefly luciferase reporter vector pGL3-basic (Promega) via NheI and HindIII sites which were designed into the forward and the reverse primers, respectively. To characterize β-catenin/TCF4 activity of various cell lines, pGL3-OT and pGL3-OF Firefly luciferase reporter plasmids with wild-type and mutant TCF4 binding sequences, respectively, were used [43]. pRL-TK (Promega), a Renilla luciferase internal control vector, was used for normalization of Firefly luciferase signals. CS2+/β-catenin-4m plasmid (quadruple-mutant with S33A, S37A, T41A and S45A mutations) and LNCX-Wnt1 and LNCX-Wnt3a plasmids were kind gifts of Prof. Xi He, Children's Hospital, Harvard Medical School (Boston, MA, USA). pcDNA-myc-hTCF4 and pcDNA-myc-ΔN-hTCF4 were from Prof. Bert Vogelstein, Johns Hopkins Oncology Center (Baltimore, MD, USA).

### Transfection and Luciferase assays

0.5 µg (per well of 6-well plate) of either pcDNA3 or pcDNA3-S33Y were transfected into Huh7 cells by using Lipofectamine 2000 reagent (Invitrogen), following the manufacturer's instructions. (For luciferase assays, pGL3-OT, pGL3-OF and pRL-TK were co-transfected). Cells were harvested at indicated time points into 1X Triton-HEPES Lysis Buffer [1% Triton X-100, 25 mM HEPES, pH 7.4, 15 mM MgSO4, 4 mM EGTA, 1 mM DTT] and frozen at −80°C. Luciferase activities were measured using Dual Glo™ Luciferase Assay System (Promega), as per the procedure provided by the manufacturer.

### RNA Extraction and RT-PCR

Cells were harvested into 350 µL of Buffer RLT and frozen at −80°C at indicated time points. Total RNA was isolated using the RNeasy® Mini Kit (Qiagen) according to the manufacturer's protocol. RNA was reverse transcribed using ImProm-II™ Reverse Transcription System (Promega) as per manufacturer's instructions RNA extraction was performed from 30 mg head tissue of F1 generation flies using the Rneasy Mini Kit (Qiagen) according to the manufacturer's protocol. RNA samples were reverse transcribed using the First Strand cDNA Synthesis Kit (Fermentas) according to the manufacturer's instructions and used as template in RT-PCRs. For Drosophila experiments, total RNA was isolated from head tissues of Drosophila fly lines. RT-PCR was performed in a total volume of 25 µl containing 50 ng of genomic DNA and 0.25 mM primers in the presence of 1.5 mM MgCl2, 0.25 mM of each dNTP and 0.5 U Taq DNA polymerase (Fermentase). The following PCR conditions were used: 5 min at 94°C; 25 cycles of 30 s at 94°C, 30 s at 57°C and 30 s at 72°C, followed by a final extension of 5 min at 72°C. The amplified PCR products were analyzed on a 1,5% agarose gel containing ethidium bromide. qRT-PCR was performed as described previously [25].

### Lithium treatment

Cells were seeded into 6-well plates and at ∼80% confluence, lithium acetate was added to a final concentration of 25 mM. Cells were harvested at indicated timepoints by adding 350 µL of RLT lysis buffer RNeasy® Mini Kit (Qiagen) and total RNA was isolated using the RNeasy® Mini Kit (Qiagen) according to the manufacturer's protocol. For Western Blotting analysis, the above procedure was repeated, but the cells were harvested at indicated timepoints by adding 700 µL of 1X Triton-HEPES Lysis Buffer [1% Triton X-100, 25 mM HEPES, pH 7.4, 15 mM MgSO_4_, 4 mM EGTA, 1 mM DTT]. The samples were passed through syringe needle (23G) and analyzed by Bradford assay for total protein levels. Equal protein amounts (100 µg) were resolved on 10% SDS-PAGE and analyzed by Western Blotting using monoclonal anti-β-catenin antibody 9E10 (a gift by M. Ozturk, Bilkent University, Ankara, Turkey) and anti-α-actin polyclonal antibody (Cell Signalling Biotech.).

### Chromatin ImmunoPrecipitation

The procedure was adapted from a previously published protocol [25]. Cells in 150 mm plates were crosslinked by adding 0.8 ml of 37% formaldehyde into 20 ml medium and incubating at 25°C for 15 min. To quench the crosslinking, 1.1 ml of 2.5 M glycine were added and incubated at 25°C for 5 min. Cells were harvested by scraping and centrifuged at 1000×g for 5 min at 4°C. The cell pellet was washed 2 times with 10 ml ice-cold PBS+1 mM PMSF. The cell pellet was resuspended in 2.5 ml of ice-cold IP buffer (150 mM NaCl, 5 mM EDTA, 1% Triton X-100, 0.5% NP-40, 50 mM Tris-HCl (pH 7.5) and 0.5 mM DTT) containing protease inhibitor cocktail (Sigma) and an extra 1 mM of PMSF. The cells were sonicated three times for 30 seconds at power setting 5 with 50% power efficiency. The debris was removed by centrifugation at 14000×g for 10 min at 4°C. 100 ml of the supernatant was saved as “input”. The rest of the supernatant was pre-cleared by incubating with 75 µL of 50% slurry of Protein A-agarose plus Salmon Sperm DNA (33 µg/ml) for 30 min at 4°C. The supernatant was incubated with anti-β-catenin monoclonal antibody (9E10) or a control antibody (Goat Anti-Rabbit/HRP) at 0.1 mg/ml concentration, overnight at 4°C. Then, 30 µL of Protein A-agarose beads were added and incubated at 4°C for 1.5 hours with gentle agitation. The beads then were washed with 1 ml of the following buffers for 5 min at 4°C: (1) Low Salt Buffer (0.1% SDS, 1% Triton X-100, 2 mM EDTA, 20 mM Tris-HCl, pH 8.1, 150 mM NaCl); (2) High Salt Buffer (0.1% SDS, 1% Triton X-100, 2 mM EDTA, 20 mM Tris-HCl, pH 8.1, 500 mM NaCl); (3) TE Buffer (10 mM Tris-HCl, 1 mM EDTA, pH 8.0). DNA was eluted twice by adding 250 µL of Elution Buffer (1% SDS and 0.1 M NaHCO_3_) and incubating at 25°C for 15 min with frequent vortexing. The two 250 µL elutions were then combined, 20 µL of 5 M NaCl was added and incubated at 65°C for 4 hours to reverse the crosslinking. Next, 10 µL of 0.5 M EDTA, 20 µL of 1 M Tris-HCl, pH 6.5 and 2 µL of 10 mg/ml proteinase K were added and the mixture was incubated for 1 hr at 45°C. DNA was extracted with traditional phenol-chloroform-isoamyl alcohol extraction and precipitated with ethanol. The DNA pellet was resuspended in 50 µL of 10 mM Tris-HCl, pH 7.4. PCR was done using a primer pair amplifying −620…+1 region of the h*MENA* promoter at the following final concentrations of reactants and cycling conditions: MgCl_2_ (2 mM), dNTP (0.5 mM), DMSO (5%), Taq DNA polymerase (Fermentas) (0.04 u/µL); 92°C 5 min, 40 cycles [92°C 30 sec, 55°C 1 min, 70°C 2 min], 70°C 5 min; Hot-start at 92°C.

### Drosophila Lines

Fly strains used were “ey-GAL4, GS88A8, UAS-Dl/Cyo” (eyeful flies), “ey-GAL4, UAS-Dl/Cyo” (sensitized flies), “UAS-whiteRNAi” (kind gifts from Bassem Hassan), “UAS-AxinRNAi”, “UAS-EnaRNAi” (obtained from Vienna Drosophila RNAi Center). All flies were raised at 18°C and all crosses were stocked at 25°C on standard fly food. Eye specific promoter eyeless was used to drive Ena RNAi expression. The eyeful flies with the downregulated Ena expression were selected from F1 generation using specific balancer markers in order to analyze the tumor and metastasis prevalence. As a negative control, *white* gene was knocked-down and as a positive control, Axin was knocked-down. To analyze the tumor prevalence, 180 flies were examined and each eye was scored separately. Eyes were counted as tumorous when the eye showed at least one fold, and metastasis could be seen as masses of amorphous red-pigmented cells outside of the eye field.

### Mouse work

C57BL/6 mice were used in this study, which was approved by the Bogazici University Ethics Committee. Mice were culled by cervical dislocation and tissues were frozen in liquid nitrogen until further use. For ChIP experiments, procedure described in the “Chromatin ImmunoPrecipitation” section was applied to the brain and liver tissues.

### In silico analysis and statistics

Promoter sequences were analyzed using Transcriptional Regulatory Element Database located at Cold Spring Harbor Laboratory website (http://rulai.cshl.edu/cgi-bin/TRED/tred.cgi?process=home) and TFexplorer (http://www.tfexplorer.org) [44]. First 2000 bp upstream of predicted transcriptional start site were analyzed. Each experiment was performed at least twice. All data are shown as means. Error bars represent standard deviations. Statistical significances were tested by Student's t-test. *P*-values less than 0.05 were considered significant (one stars) and *p*-values less than 0.005 were considered very significant (two stars).

## References

[1] Cadigan KM, Nusse R (1997). Wnt signaling: a common theme in animal development.. Genes Dev.

[2] Peifer M, Polakis P (2000). Wnt signaling in oncogenesis and embryogenesis–a look outside the nucleus.. Science.

[3] Polakis P (2000). Genes Dev.

[4] Stambolic V, Ruel L, Woodgett JR (1996). Lithium inhibits glycogen synthase kinase-3 activity and mimics wingless signalling in intact cells.. Curr Biol.

[5] Behrens J, Jerchow BA, Wurtele M, Grimm J, Asbrand C (1998). Functional interaction of an axin homolog, conductin, with beta-catenin, APC, and GSK3beta.. Science.

[6] Behrens J, Jerchow BA, Wurtele M, Grimm J, Asbrand C (1998). Functional interaction of an axin homolog, conductin, with beta-catenin, APC, and GSK3beta.. Science.

[7] Hart MJ, de los Santos R, Albert IN, Rubinfeld B, Polakis P (1998). Downregulation of beta-catenin by human Axin and its association with the APC tumor suppressor, beta-catenin and GSK3 beta.. Curr Biol.

[8] Lin SY, Xia W, Wang JC, Kwong KY, Spohn B (2000). Beta-catenin, a novel prognostic marker for breast cancer: its roles in cyclin D1 expression and cancer progression.. Proc Natl Acad Sci U S A.

[9] Ryo A, Nakamura M, Wulf G, Liou YC, Lu KP (2001). Pin1 regulates turnover and subcellular localization of beta-catenin by inhibiting its interaction with APC.. Nat Cell Biol.

[10] Ugolini F, Charafe-Jauffret E, Bardou VJ, Geneix J, Adelaide J (2001). WNT pathway and mammary carcinogenesis: loss of expression of candidate tumor suppressor gene SFRP1 in most invasive carcinomas except of the medullary type.. Oncogene.

[11] Wissmann C, Wild PJ, Kaiser S, Roepcke S, Stoehr R (2003). WIF1, a component of the Wnt pathway, is down-regulated in prostate, breast, lung, and bladder cancer.. J Pathol.

[12] Hoschuetzky H, Aberle H, Kemler R (1994). Beta-catenin mediates the interaction of the cadherin-catenin complex with epidermal growth factor receptor.. J Cell Biol.

[13] Shibamoto S, Hayakawa M, Takeuchi K, Hori T, Oku N (1994). Tyrosine phosphorylation of beta-catenin and plakoglobin enhanced by hepatocyte growth factor and epidermal growth factor in human carcinoma cells.. Cell Adhes Commun.

[14] Kanai Y, Ochiai A, Shibata T, Oyama T, Ushijima S (1995). c-erbB-2 gene product directly associates with beta-catenin and plakoglobin.. Biochem Biophys Res Commun.

[15] Adam L, Vadlamudi RK, McCrea P, Kumar R (2001). Tiam1 overexpression potentiates heregulin-induced lymphoid enhancer factor-1/beta -catenin nuclear signaling in breast cancer cells by modulating the intercellular stability.. J Biol Chem.

[16] Schroeder JA, Adriance MC, McConnell EJ, Thompson MC, Pockaj B (2002). ErbB-beta-catenin complexes are associated with human infiltrating ductal breast and murine mammary tumor virus (MMTV)-Wnt-1 and MMTV-c-Neu transgenic carcinomas.. J Biol Chem.

[17] Playford MP, Bicknell D, Bodmer WF, Macaulay VM (2000). Insulin-like growth factor 1 regulates the location, stability, and transcriptional activity of beta-catenin.. Proc Natl Acad Sci U S A.

[18] Danilkovitch-Miagkova A, Miagkov A, Skeel A, Nakaigawa N, Zbar B (2001). Oncogenic mutants of RON and MET receptor tyrosine kinases cause activation of the beta-catenin pathway.. Mol Cell Biol.

[19] Kim K, Lee KY (2001). Tyrosine phosphorylation translocates beta-catenin from cell-cell interface to the cytoplasm, but does not significantly enhance the LEF-1-dependent transactivating function.. Cell Biol Int.

[20] Arteaga CL (2002). Overview of epidermal growth factor receptor biology and its role as a therapeutic target in human neoplasia.. Semin Oncol.

[21] Normanno N, Bianco C, De Luca A, Maiello MR, Salomon DS (2003). Target-based agents against ErbB receptors and their ligands: a novel approach to cancer treatment.. Endocr Relat Cancer.

[22] Pawlak G, Helfman DM (2001). Cytoskeletal changes in cell transformation and tumorigenesis.. Curr Opin Genet Dev.

[23] Kwiatkowski AV, Gertler FB, Loureiro JJ (2003). Function and regulation of Ena/VASP proteins.. Trends Cell Biol.

[24] Di Modugno F, Bronzi G, Scanlan MJ, Del Bello D, Cascioli S (2004). Human Mena protein, a serex-defined antigen overexpressed in breast cancer eliciting both humoral and CD8+ T-cell immune response.. Int J Cancer.

[25] Kavak E, Najafov A, Ozturk N, Seker T, Cavusoglu K (2010). Cell Signal.

[26] Morin PJ, Sparks AB, Korinek V, Barker N, Clevers H (1997). Activation of beta-catenin-Tcf signaling in colon cancer by mutations in beta-catenin or APC.. Science.

[27] Cha MY, Kim CM, Park YM, Ryu WS (2004). Hepatitis B virus X protein is essential for the activation of Wnt/beta-catenin signaling in hepatoma cells.. Hepatology.

[28] Gurzu S, Jung I, Prantner I, Ember I, Pavai Z (2008). The expression of cytoskeleton regulatory protein Mena in colorectal lesions.. Rom J Morphol Embryol.

[29] Toyoda A, Kawana H, Azuhata K, Yu J, Omata A (2009). Aberrant expression of human ortholog of mammalian enabled (hMena) in human colorectal carcinomas: implications for its role in tumor progression.. Int J Oncol.

[30] Tao W, Pennica D, Xu L, Kalejta RF, Levine AJ (2001). Wrch-1, a novel member of the Rho gene family that is regulated by Wnt-1.. Genes Dev.

[31] Blair IP, Chetcuti AF, Badenhop RF, Scimone A, Moses MJ (2006). Positional cloning, association analysis and expression studies provide convergent evidence that the cadherin gene FAT contains a bipolar disorder susceptibility allele.. Mol Psychiatry.

[32] Menzies AS, Aszodi A, Williams SE, Pfeifer A, Wehman AM (2004). Mena and vasodilator-stimulated phosphoprotein are required for multiple actin-dependent processes that shape the vertebrate nervous system.. J Neurosci.

[33] Lindwall C, Fothergill T, Richards LJ (2007). Commissure formation in the mammalian forebrain.. Curr Opin Neurobiol.

[34] Kahler AK, Djurovic S, Kulle B, Jonsson EG, Agartz I (2008). Association analysis of schizophrenia on 18 genes involved in neuronal migration: MDGA1 as a new susceptibility gene.. Am J Med Genet B Neuropsychiatr Genet.

[35] Katsu T, Ujike H, Nakano T, Tanaka Y, Nomura A (2003). The human frizzled-3 (FZD3) gene on chromosome 8p21, a receptor gene for Wnt ligands, is associated with the susceptibility to schizophrenia.. Neurosci Lett.

[36] Miyaoka T, Seno H, Ishino H (1999). Increased expression of Wnt-1 in schizophrenic brains.. Schizophr Res.

[37] Yang SD, Yu JS, Lee TT, Yang CC, Ni MH (1995). Dysfunction of protein kinase FA/GSK-3 alpha in lymphocytes of patients with schizophrenic disorder.. J Cell Biochem.

[38] Bottomly D, Kyler SL, McWeeney SK, Yochum GS (2010). Identification of {beta}-catenin binding regions in colon cancer cells using ChIP-Seq.. Nucleic Acids Res.

[39] Nicolas M, Wolfer A, Raj K, Kummer JA, Mill P (2003). Notch1 functions as a tumor suppressor in mouse skin.. Nat Genet.

[40] Reya T, Clevers H (2005). Wnt signalling in stem cells and cancer.. Nature.

[41] Di Modugno F, DeMonte L, Balsamo M, Bronzi G, Nicotra MR (2007). Molecular cloning of hMena (ENAH) and its splice variant hMena+11a: epidermal growth factor increases their expression and stimulates hMena+11a phosphorylation in breast cancer cell lines.. Cancer Res.

[42] Pino MS, Balsamo M, Di Modugno F, Mottolese M, Alessio M (2008). Human Mena+11a isoform serves as a marker of epithelial phenotype and sensitivity to epidermal growth factor receptor inhibition in human pancreatic cancer cell lines.. Clin Cancer Res.

[43] He TC, Sparks AB, Rago C, Hermeking H, Zawel L (1998). Identification of c-MYC as a target of the APC pathway.. Science.

[44] Kim J, Seo J, Lee YS, Kim S (2005). TFExplorer: integrated analysis database for predicted transcription regulatory elements.. Bioinformatics.

